# Biological control agents colonize litchi fruit during storage and stimulate physiological responses to delay pericarp browning

**DOI:** 10.3389/fmicb.2022.1093699

**Published:** 2023-01-05

**Authors:** Li Zheng, Shilian Huang, Jiehao Huang, Yizhen Deng, Zhenxian Wu, Zide Jiang, Guohui Yu

**Affiliations:** ^1^Key Laboratory of Green Prevention and Control on Fruits and Vegetables in South China, Ministry of Agriculture and Rural Affairs, Guangdong University Key Laboratory for Sustainable Control of Fruit and Vegetable Diseases and Pests, Innovative Institute for Plant Health, College of Agriculture and Biology, Zhongkai University of Agriculture and Engineering, Guangzhou, China; ^2^Guangdong Province Key Laboratory of Microbial Signals and Disease Control, Department of Plant Pathology, South China Agricultural University, Guangzhou, China; ^3^Key Laboratory of South Subtropical Fruit Biology and Genetic Resource Utilization, Ministry of Agriculture and Rural Affairs, Guangdong Provincial Key Laboratory of Tropical and Subtropical Fruit Tree Research, Institute of Fruit Tree Research, Guangdong Academy of Agricultural Sciences, Guangzhou, China; ^4^Guangdong Provincial Key Laboratory of Postharvest Science of Fruit and Vegetables, College of Horticulture, Engineering Research Center for Postharvest Technology of Horticultural Crops in South China, South China Agricultural University, Ministry of Education, Guangzhou, China

**Keywords:** anthocyanin content, biocontrol agents, colonization, delayed browning, enzyme activity, litchi fruit

## Abstract

**Introduction:**

Litchi is an economically important fruit in subtropical countries, but pericarp browning can limit its shelf life outside of controlled storage conditions. Effective and sustainable biological control strategies are needed to protect fruit against postharvest browning.

**Results and Discussion:**

In this study, we show that the four bacterial strains *Bacillus licheniformis* HS10, *B. amyloliquefaciens* LI24 and PP19, and *Exiguobacterium acetylicum* SI17 can delay fruit browning in both laboratory trials (LTs) and field plus laboratory trials (FLTs). Strains HS10, LI24, PP19 and SI17 showed 47.74%, 35.39%, 33.58% and 32.53% browning-inhibitory efficacy respectively at 180 h in LT. Litchi sarcocarp interior sourced isolate SI17 showed 74.05% inhibit-brown efficacy at 216 h in FLTs, performing better in FLT than in LT. Furthermore, strains PP19 and SI17 colonized the fruit pericarp and increased total phenolic and anthocyanin contents but decreased peroxidase and polyphenol oxidase activity. This is the first report of *E. acetylicum* (SI17) and *B. licheniformis* (HS10) strains acting as biological control agents (BCAs) to delay postharvest browning in litchi fruit. We conclude that PP19 and SI17 are promising BCAs against fruit browning, and their application could be effective for prolonging the shelf life of harvested litchi fruit.

## Introduction

Litchi (*Litchi chinensis* Sonn.) is grown widely as a commercial crop in subtropical Asia, South Africa, Australia, the United States (especially Hawaii), and Israel (Jiang et al., 2001). In China, litchi is mainly cultivated in the southern provinces including Guangdong, Guangxi, Fujian, and Hainan. Litchi is one of the most perishable subtropical fruits but deteriorates rapidly due to physiological browning of the pericarp and postharvest diseases caused by the fungi *Peronophythora litchii* and *Colletotrichum* spp. (ref). Under typical commercial conditions, synthetic fungicides such as prochloraz, dimethomorph, and sulfur dioxide preservative are applied to extend postharvest litchi storage. However, the application of fungicides or preservatives is not environment-friendly, potentially threatening food safety. So, alternative but effective methods for post-harvested litchi fruit preservation are needed. Various means to control postharvest decay in litchi have been investigated, including biological control agents (BCAs), temperature management, heat treatments, various packaging options, and controlled atmosphere storage (Jiang and Joyce, 2003). Of these options, BCAs have the greatest potential in delaying pericarp browning as chemical fungicides.

Browning of the litchi fruit pericarp is a visible manifestation of phenolic compound oxidation catalyzed by peroxidase (POD) and polyphenol oxidase (PPO) and/or the degradation of anthocyanins (Zhang et al., 2001; Jiang et al., 2004; Neog and Saikia, 2010). Spraying a suspension of the bacterial strain *Lactobacillus plantarum* onto litchi fruit maintains high anthocyanin and phenolic contents in the peel under laboratory conditions (Martínez-Castellanos et al., 2011). Early application of BCAs in the field facilitates bacterial colonization to litchi pericarp, and thus delay fruit browning after harvest (Smilanick et al., 1993). Early establishment of a BCA in the pericarp before fruits reach full maturity is especially important to protect against rapid decay and to enhance the concentration of *trans*-resveratrol (Xu et al., 2018) and anthocyanins (Iriti et al., 2004). Therefore, the regulation of physiological processes in harvested litchi fruit could reduce pericarp browning and extend the shelf life of harvested litchi.

Biological control agents often exert biocontrol activity in a tissue-dependent manner, performing best in its source tissues. Berg et al. (2001) found most of the tested rhizobacterial strains promoted the growth of strawberry seeds. Dutta and Thakur (2017) reported that region-specific tea-associated microorganisms are important in establishing integrated nutrient management and integrated disease management strategies. Additionally, BCAs isolated from specific tissues can be used to manage diseases in those same tissues (Manso and Nunes, 2011) since they are often better adapted to living in those environments than the pathogens themselves.

In this study, we selected four bacterial strains from different sources: *Bacillus licheniformis* HS10 (rhizospheric soil of healthy cucumber, Zheng et al., 2019), *Bacillus amyloliquefaciens* LI24 (from litchi leaf interior) and PP19 (from litchi pericarp exterior), and *Exiguobacterium acetylicum* SI17 (from litchi sarcocarp interior). To explore whether the BCAs have a greater effect on browning inhibition, we examined their effectiveness in reducing postharvest browning and prolonging the shelf life of litchi fruit under two different conditions. We also investigated the potential mechanisms by which BCAs delay browning. The results from this study should contribute to preventing the postharvest decay of litchi fruit.

## Materials and methods

### Culture of bacterial isolates

Four bacterial cultures (*B. amyloliquefaciens* PP19 and LI24, *E. acetylicum* SI17, and *B. licheniformis* HS10), stored in the Fungus Laboratory of the Department of Plant Pathology, South China Agricultural University (SCAU), were grown in Luria-Bertani (LB) medium (tryptone 10.0 gL^−1^, yeast extract 5 gL^−1^, NaCl 10.0 gL^−1^, pH 7.0) at 28°C with shaking at 200 rpm for 24 h. The resulting bacterial suspensions were adjusted to 5 × 10^8^ colony-forming units (CFUs) mL^−1^ with sterile water, and the titer of the suspensions was confirmed by plating in conjunction with measuring their optical density using a spectrophotometer at 600 nm. The suspensions were used for further tests.

### Measurement of browning inhibition by the four bacterial strains in LTs

The trial was conducted to assess bacterial inhibition of fruit browning with postharvest application of laboratory trials (LTs). In LTs, the healthy fruit of litchi were harvested and immediately transported to the laboratory for processing. Every 30 pieces of detached fruit were placed in a plastic container (323 mm × 220 mm × 100 mm), in which the bottom was covered with two pieces of sterile filter paper (*D* = 18 cm), moistened with 15 ml sterile water. Fruit were sprayed with a bacterial culture broth at 5 × 10^7^ CFUs mL^−1^, which was 10-fold diluted from the initial bacterial culture with sterile water, two control treatments with the chemical antioxidant/preservative butylated hydroxytoluene (BHT, 1 mM, Shanghai Lingfeng chemical reagent CO. LTD, CAS No. 128–37-0), and 1/10 strength LB medium (non-treated control). Every treatment was sprayed onto the fruits at the 80–85% ripening degree, there were three replicates per treatment, with 30 pieces of fruit per replicate. The relative humidity in the container was 85–90%, which was placed in a small greenhouse maintained at 25°C and with a 12-h-light/12-h-dark cycle and the relative humidity of 60–75% (parameters were monitored by the TH6 automatic humidity and temperature data logge, Hangzhou Meacon Automation Technology Co., Ltd.). The experiment was performed twice independently on cv. ‘Feizixiao’ in Danzhou (LT2017-1, Nanyang farm, Nada Township, Danzhou City, Hainan Province, fruits about 80% ripe) and Guangzhou (LT2017-2, Taiping Township, Conghua district, Guangzhou City, Guangdong Province, fruits about 85% ripe).

Browning development was monitored during 84–192 h after treatment. Browning severity was as defined by Jiang et al. (2001) with minor modification of the scales for brown areas on each fruit surface: 0, 0% browning; 1, ≤5% browning; 3, 6–10% browning; 5, 11–25% browning; 7, 26–50% browning; and 9, >50% browning. Browning index and browning inhibition were calculated as follows: browning index = [*Σ* (browning level × number of fruit at each level)/(the highest level × total number of fruit)]; browning inhibition (%) = [(browning index of control - browning index of treatment)/browning index of control] × 100.

### Determination of the efficacy of browning inhibition by the four bacterial strains in FLTs

With the preharvest application of FLT2017-1 (a field plus laboratory trial), four strains individually (5 × 10^7^ CFUs mL^−1^) or one of two controls (as described for LT2017-1) were applied to the on-tree fruit of litchi cultivar ‘Feizixiao’ at about 65% ripening degree in the field by spraying. One tree was used for each treatment, with fruit growing in different parts of the tree being considered different replicates. At least 300 fruits per treatment were pretreated in the field, but only 90 were selected for processing in the laboratory (30 fruits per replicate). When pretreated in the field, fruits were about 65% ripe. At 7 days post treatment, the pretreated fruits (having reached 80% ripeness) in all treatments were harvested and immediately transported to the laboratory. Subsequent experimental conditions and the determination of browning severity were as described above for LTs.

### Bacterial colonization of the fruit pericarp

Previously identified spontaneous rifampicin-resistant mutants of wild-type strains PP19, SI17, LI24, and HS10 are referred to as PP19rif, SI17rif, LI24rif, and HS10rif, respectively. These rif strains were cultured under routine conditions in LB medium containing 100 mg L^−1^ rifampicin. The resulting bacterial cell suspensions were adjusted to 5 × 10^7^ CFUs mL^−1^ with sterile 0.85% (w/v) NaCl. Mock-treated fruit served as a control (sterile 0.85% NaCl). Tween-20 was added to the cell suspensions at a final concentration of 0.01% (v/v) prior to inoculation by spraying in the field.

Fruits (65% ripe, cv. ‘Feizixiao’, May 2017) were treated with four bacteria (rifPP19, rifSI17, rifPI26, and rifHS10) in the field. Fruit treated with sterile 0.85% NaCl served as the non-treated control. After 7 days, 90 pieces of fruit per treatment at about 80–85% ripeness were brought back to the lab and placed in lidded containers, sprayed again with the bacterial cell suspension of the same isolate, and incubated at 25°C. At 24, 48, and 72 hpt (hours post treatment), six pieces of fruit per time point per treatment were collected, and bacteria were immediately isolated from the pericarp as described by Yang et al. (2008). Colonies on each plate of rifampicin-amended medium were counted to determine the population size of each tested mutant in the pericarp. This trial was repeated with fruit of litchi cultivar ‘Huaizhi’ (July 2017, Taiwan farm, Huadong Township, Guangzhou City, Guangdong Province). For the experiment on Huaizhi, only three bacterial treatments (rifPP19, rifSI17, and rifHS10) were included, and their population sizes in pericarps were determined at 48, 72, and 96 hpt.

### Enzymatic activity assay and measurement of relevant compounds in the pericarp

Samples were treated with the isolates PP19 and SI17 as follows: one of the two strains (5 × 10^7^ CFUs mL^−1^) or LB medium (mock-treated control) was sprayed onto litchi fruits. The samples were collected from the same batch of fruit as used in LT2017-1. Three fruits for each of the three replicates (a total of nine fruits per treatment) were harvested at 0, 24, 36, 48, 60, 72, and 84 h after treatment. The pericarp was immediately packed in tin foil, frozen in liquid nitrogen for 30 min, and stored at −80°C. All enzyme extraction procedures were conducted at 4°C using a homogenizer (Precellys Evolution, Bertin, France). The preservation-related enzymes and relevant compounds being assessed included the following: antioxidant enzymes PPO (EC 1.10.3.2) and POD (EC 1.11.1.7), the anthocyanase anthocyanin-β-glucosidase (ANT, EC 3.2.1.21), anthocyanins, and total phenolic contents (TPCs). PPO and POD activity was assessed with a detection kit (Nanjing Jiancheng Biological Engineering Institute, Nanjing, China). The assay for TPC used the Folin–Ciocalteu reagent (Ainsworth and Gillespie, 2007). Anthocyanin concentration and ANT activity in the pericarp were measured as described by Zhang et al. (2001).

### Data analysis

Data on fruit browning index, browning inhibition, colonization ability, enzymatic activities, and concentrations of relevant compounds in the pericarp were analyzed at the factorial level by analysis of variance, and significant (*p* < 0.05) differences among treatments were determined using a statistical software data processing system (DPS version 7.05, Zhejiang University, Hangzhou, China). When the results of F-tests were significant at *p* < 0.05, mean values were compared according to the least significant difference test. In LTs for browning inhibition, enzyme activity, and concentrations of relevant compounds as well as the three FLTs (one efficacy assay and two colonization assays), the only variable was bacterial treatment and the fixed factor was the method of applying bacteria. The enzymatic activities and concentrations of relevant compounds in the pericarp were expressed on a fresh weight basis.

## Results

### Inhibition of fruit browning by BCAs

**Figure 1:**
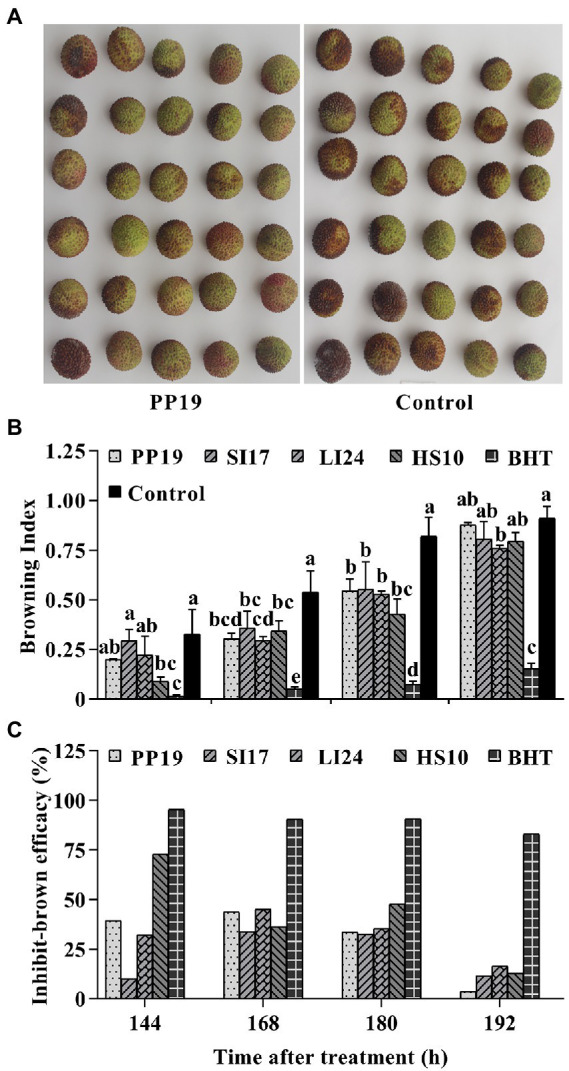
Browning inhibition efficacy of four strains on litchi fruit. Bacteria (5 × 10^7^ CFUs mL^−1^) were sprayed on cv. ‘Feizixiao’ (75–80% ripeness, Danzhou, Hainan province) in a laboratory. The images display a comparison of browning indices between the litchi fruit treated with PP19 and LB broth (Control) at 144 h **(A)**. Bar charts displaying the browning indices of harvested litchi fruit treated with the four strains PP19, SI17, LI24, and HS10, and the BHT and LB broth controls **(B)**. The browning inhibition efficacies are shown in panel **(C)**. Data are presented as means of replicates ± standard errors; different letters indicate significant differences between treatments according to LSD test at *p* < 0.05.

**Figure 2:**
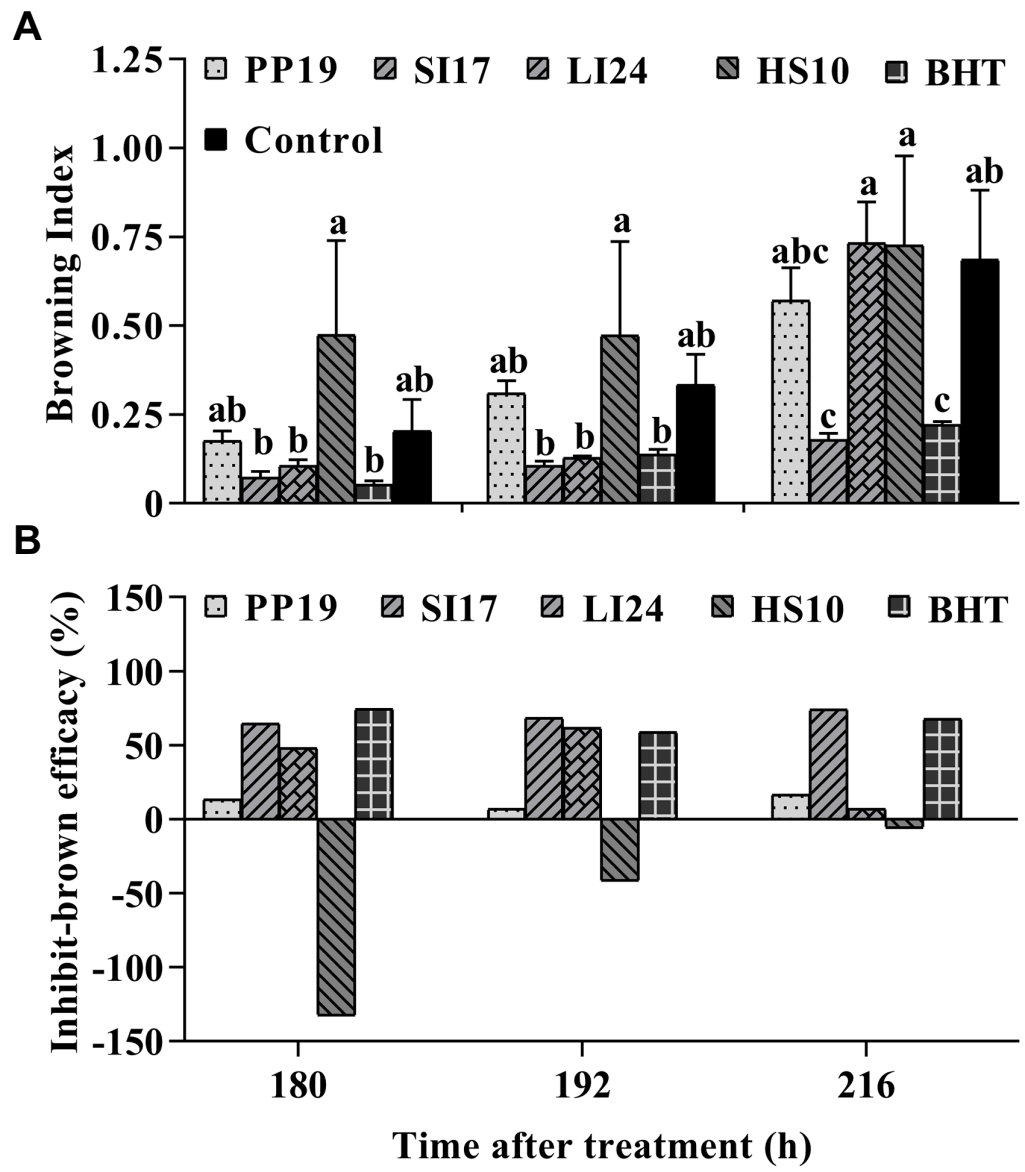
The efficacy of representative bacterial strains against fruit browning in FLT2017-1. Bacteria (5 × 10^7^ CFUs mL^−1^) were sprayed on cv. ‘Feizixiao’ (65% ripeness, Danzhou, Hainan Province) in the field, and fruits (about 80% ripeness) were picked and placed in a preserving box in the laboratory after 7 days. Browning indices of preharvest litchi fruit treated with the four strains PP19, SI17, LI24, and HS10, and the BHT and LB broth controls **(A)**. The browning inhibition efficacies are shown in panel **(B)**. Data are presented as means of replicates ± standard errors; different letters indicate significant differences between treatments according to LSD test at *p* < 0.05.

We assessed the efficacy of BCAs in inhibiting fruit browning in both LTs and FLT. Litchi fruit are usually bright red in color at harvest (Figure 1A). In LTs, pericarp browning increased over time in the control group (Figures 1B, 2; Supplementary Figure S1). PP19, SI17, LI24, HS10 and BHT treatment reduced browning index of litchi fruit than the untreated fruit (control), at both 168 and 180 h. However, BCA treatments led to higher levels of browning than BHT treatment at all four time points. There was no difference between BCA treatments and control at 192 h, except for the treatment with LI24. In contrast, treatment with PP19, SI17, LI24, or HS10 lowered the browning index at least at two time points (Figure 1B), with corresponding browning delay efficacies approaching 43.7, 33.6, 45.1, and 36.4%, respectively, at 168 h (Figure 1C).

In FLTs, strain SI17 and the chemical agent BHT decreased the browning index at least at one time point, while strains PP19, HS10, and LI24 failed to suppress fruit browning in the field (Figure 2A; Supplementary Table S1). Among BCAs, SI17 led to the highest browning-inhibitory efficacy, having a similar effect on browning as BHT treatment. HS10 treatment led to more browning than the control treatment, therefore displaying a negative browning inhibition efficacy (Figure 2B). BHT and SI17 treatments consistently reduced fruit browning indices in all three trials (LT2017-1, LT2017-2, and FLT2017-1). While strains PP19 and LI24 consistently lowered the extent of fruit browning in both LTs, strain HS10 reduced fruit browning only in a single LT (LT2017-1).

Overall, strains PP19, SI17, LI24, and HS10 appeared promising in LTs. However, strain SI17 performed the best in FLT, with an ARE >55% (Supplementary Table S2). Strain SI17 performed well in both LTs and FLTs. In conclusion, preharvest inoculation of litchi fruit with BCAs could be a promising approach to delay browning, depending on the BCA used.

### Colonization competence of the isolates

**Figure 3:**
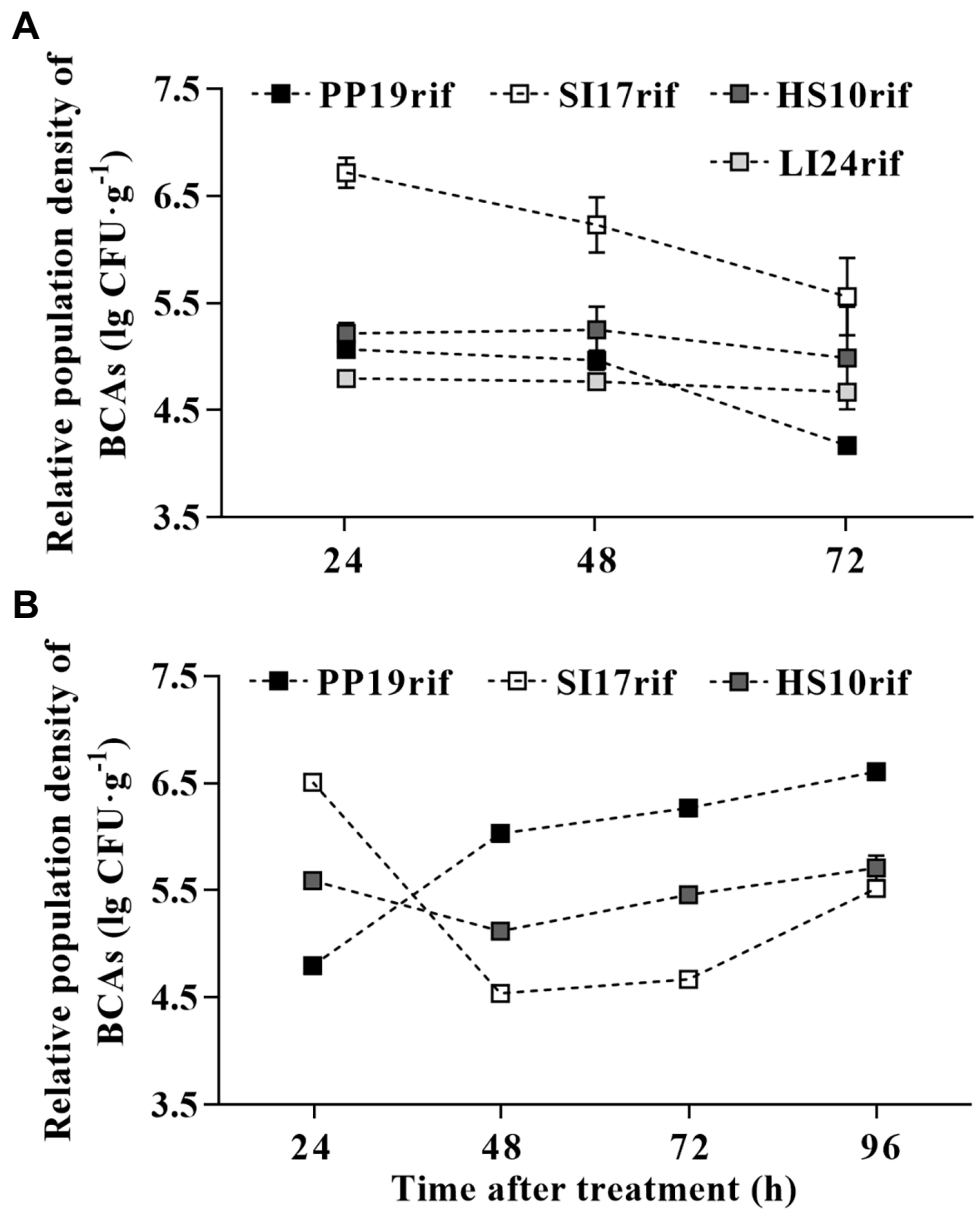
The competence of BCAs to colonize the fruit pericarp of two litchi cultivars ‘Feizixiao’ and ‘Huaizhi’ in FLT2017-2. The population densities of PP19rif, SI17rif, LI24rif, and HS10rif in ‘Feizixiao’ **(A)** and ‘Huaizhi’ **(B)** fruit pericarp over time. The fruit samples (about 65% ripeness) were sprayed with a bacterial suspension (5 × 10^7^ CFUs mL^−1^) in the field in Danzhou (Hainan province) and Guangzhou (Guangdong province). After 7 days, the fruits (about 80% or 85% ripeness) were transported to the laboratory, where they were placed in a preserving box and sprayed with the bacterial suspension (5 × 10^7^ CFUs mL^−1^) again. Data are presented as means of replicates ± standard errors; different letters indicate significant differences between treatments according to LSD test at *p* < 0.05.

To investigate the mechanism(s) by which the four BCAs inhibited browning, we evaluated their colonization competence individually. As shown in Figure 3A, all strains colonized the pericarp when sprayed onto cv. ‘Feizixiao’ fruit. We observed variation in population density, with strain SI17rif reaching the highest titer during our experiment, followed by strain HS10rif. The titer of strains HS10rif and LI24rif remained constant from 24 to 72 h, while that of PP19rif diminished from 48 to 72 h (Figure 3A). We repeated the experiment on fruit from a different litchi cultivar (‘Huaizhi’) and observed different patterns for strains SI17rif, PP19rif, and HS10rif (Figure 3B). Over time, the population density of strain PP19rif increased, while that of strain SI17rif decreased. Similar to the pattern observed in ‘Feizixiao’, the population density of HS10rif remained constant from 24 to 72 h (Figure 3B). We therefore conclude that strain SI17rif can better colonize the pericarp of cv. ‘Feizixiao’ than cv. ‘Huaizhi’, whereas the opposite is true for strain PP19rif.

### Browning-related enzymes and compounds

We selected strains PP19 and SI17 for further characterization, as they inhibited browning in LTs and FLTs most efficiently. We determined their effects on the enzymatic activity of PPO, POD, and ANT, which affect fruit browning, as well as anthocyanin content and TPC using pericarps from the litchi cv. ‘Feizixiao’ collected from the same batch used in LT2017-1.

**Figure 4:**
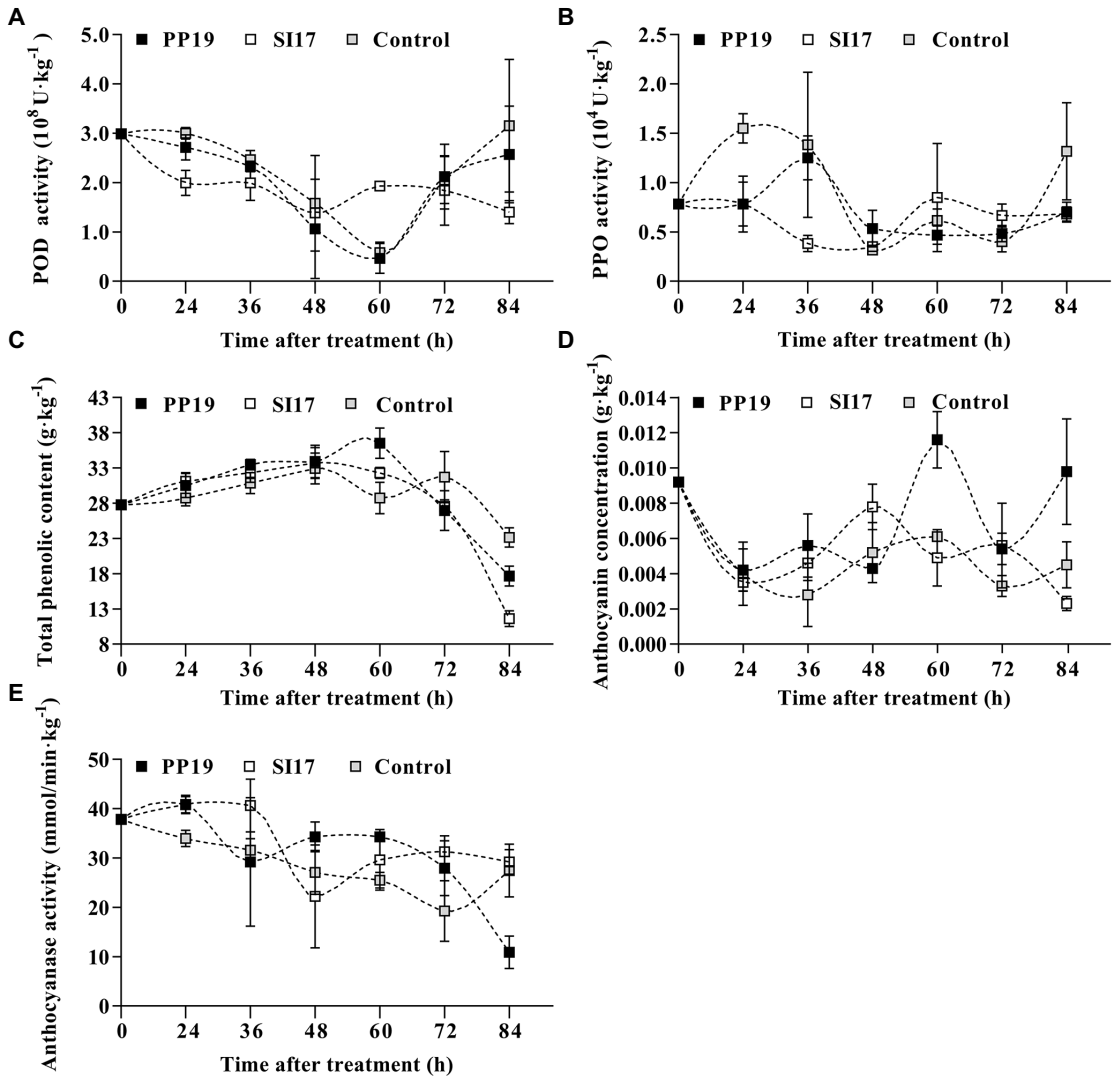
Activity or concentration changes in the litchi fruit pericarp influenced by PP19 and SI17 treatments. POD **(A)** and PPO **(B)** activity, TPC **(C)** and anthocyanin concentration **(D)**, and ANT activity **(E)**. Bacteria (5 × 10^7^ CFUs mL^−1^) were sprayed onto ‘Feizixiao’ fruit (about 80% ripeness) in LTs (Danzhou, Hainan province). Measurement was performed at 0, 24, 36, 48, 60, 72, and 84 h after treatment. Data are presented as means of replicates ± standard errors; different letters indicate significant differences between treatments according to LSD test at *p* < 0.05.

POD activity decreased from 0 to 60 h before increasing again in all samples, with peak activity reached at 84 h (control), 24 h (PP19), and 0 h (Sl17). In addition, strains SI17 and PP19 both exhibited lower POD activity than the mock-inoculated control except at 60 h (Figure 4A). PPO activity was high at first before dropping in the middle of the time course, followed by a later rise. PPO activity was highest at 24 h (control), 36 h (PP19), and 60 h (Sl17). Values measured for fruit inoculated with PP19 or SI17 were generally slightly lower than those for the control, except at 48 h for PP19 and 60–72 h for SI17 (Figure 4B). TPC of all fruit showed a similar pattern regardless of treatment, with a gradual rise until 60 h followed by a sharp drop (Figure 4C). Fruit inoculated with strains SI17 and PP19 had slightly higher TPC than control fruit from 0 to 60 h but had slightly lower levels thereafter (Figure 4C). Anthocyanin content varied in all samples over the time course, with peak accumulation at 60 h (control and PP19) or 48 h (Sl17). Anthocyanin contents were higher in fruit treated with strains PP19 or SI17 than in mock-treated control fruit except at 48, 60, and 84 h (Figure 4D). ANT activity rose then dropped in SI17 and PP19 samples, but dropped then rose in control samples, with peak activity at 0 h (control), 36 h (Sl17), and 24 h (PP19). ANT activity showed a sharp drop in PP19-inoculated fruit from 24 to 36 h and again from 72 to 84 h; likewise, SI17-inoculated fruit displayed a decrease in ANT activity from 36 to 48 h, while mock-inoculated control fruit showed a gradual decrease in ANT activity (Figure 4E). We concluded that inoculation with the strains PP19 and SI17 induces similar changes in defense-related enzyme activity and the concentration of certain compounds. In addition, we noticed that strain SI17 provoked earlier responses in litchi fruit than PP19, along with higher enzymatic activity and compound concentrations, especially from 48 to 60 h.

## Discussion

The use of microorganisms for the control of postharvest fruit decay has been actively pursued (Ippolito and Nigro, 2000) to increase overall quality retention and extend the storage life of litchi fruit. Postharvest management techniques using BCAs, including *Bacillus subtilis* (Jiang et al., 2001; Sivakumar et al., 2007, 2008), *B. amyloliquefaciens* LY-1 (Wu et al., 2017), and *Lactobacillus plantarum* LAB (Martínez-Castellanos et al., 2011), delay browning of the litchi pericarp. Our current study demonstrates that *B. amyloliquefaciens* strains LI24 and PP19 significantly inhibit fruit browning (Supplementary Table S2; Figure 1; Supplementary Figure S1), as previously reported (Wu et al., 2017). In addition, the two strains *E. acetylicum* SI17 and *B. licheniformis* HS10 also delayed fruit browning in LTs (Supplementary Table S2; Figure 1; Supplementary Figure S1), which adds to the list of promising BCA species able to inhibit postharvest fruit browning.

Source isolates are expected to display better establishment in field conditions, as they are able to colonize host plants (Deketelaere et al., 2017). The different source strains PP19, SI17, LI24, and HS10 showed potential for inhibiting postharvest litchi browning in LTs. In FLTs, however, SI17 (isolated from the litchi sarcocarp interior) had the same anti-browning effect as BHT, while HS10 (isolated from rhizospheric soil of healthy cucumber) promoted litchi browning and LI24 (isolated from the litchi leaf interior) and PP19 (isolated from the litchi pericarp exterior) showed little effect on litchi browning. These results indicate that isolates from particular sources may have better biocontrol potential in field conditions.

Aside from postharvest handling treatments, preharvest approaches allow plants to be more resistant to later abiotic and/or biotic stress (Wu et al., 2017; Dukare et al., 2018). In our results, litchi trees pretreated in the field with strain SI17 or BHT displayed a substantial delay in natural fruit decay, color changes in the pericarp, and loss of firmness after harvest (Supplementary Table S2; Figure 2). These observations were consistent with results obtained when spraying natural or synergistic compounds (such as putrescine, salicylic acid, paclobutrazol, oligochitosan, and kojic acid) and/or BCAs (such as *Cryptococcus laurentii* combined with chitosan) before harvest, which also contributed to better storage control and quality maintenance (Huang et al., 2008; Meng and Tian, 2009; Benkeblia et al., 2011; Yan et al., 2012; Shah et al., 2017). Therefore, preharvest treatment may be regarded as a promising technology with applications in fruit storage, as it appears to maintain fruit quality and reduce storage losses of horticultural products.

According to Ippolito and Nigro (2000), the timing of pre- and/or postharvest elicitor treatments and the developmental stage of the fruit may be important in determining the efficacy of postharvest decay and browning, as do adherence of the treatments to the fruit surface, treatment compatibility, and survival of the plant in variable environments (Meng and Tian, 2009; Dukare et al., 2018). In FLTs and LTs, inoculation with strains SI17, PP19, LI24, and HS10 decreased the browning index (Figures 1, 2; Supplementary Figure S1), which indicated that these BCAs colonized the fruit pericarp. Our study confirmed speculation that the four BCAs used here can colonize the litchi pericarp in the two cvs. ‘Feizixiao’ and ‘Huaizhi’ (Figure 3). Cai et al. (2008) and Wu et al. (2017) reported that effective BCAs can colonize the epidermal cells of litchi fruit and observed that the extent of colonization positively correlates with their preservation effects. Strain SI17 performed better in FLTs than in LTs (Figure 1; Supplementary Table S2), perhaps due to better colonization in cv. ‘Feizixiao’ under these conditions (Figure 3A). Strain Sl17 is an endophytic bacterium that colonizes the interior of healthy sarcocarps, and it showed better adaptability to various field conditions. In addition, we also established that strain PP19 inhibits browning more effectively in cv. ‘Huaizhi’ (Supplementary Figure S1) than in cv. ‘Feizixiao’ in LTs (Figure 1), suggesting cultivar-specific fruit colonization dynamics of PP19 (Figure 2B).

Based on our findings, we propose that preharvest spraying with strain SI17 should be combined with postharvest treatment with PP19 or BHT for maximal protection against browning. This treatment would be similar to that presented by Meng et al. (2008), which consisted of a preharvest chitosan spray, a postharvest chitosan coating, or both to protect fruit against decay. In conclusion, this promising BCA (strain Sl17) appears to occupy the fruit niche and proliferate rapidly when fruit is at the harvest stage. Strain Sl17 also colonized the fruit pericarp better than the other bacterial strains tested. We hypothesize that a portion of a plant’s defense against fruit browning may be attributable to the native microflora and the composition of beneficial BCAs on the pericarp (Wang et al., 2017; Dukare et al., 2018).

Enzymatic browning in litchi may involve an ANT-anthocyanin-TPC-PPO-POD reaction (Jiang et al., 2004; Wang et al., 2010). We detected lower PPO and POD activity but higher TPC in fruit treated with strains SI17 and PP19 from 0 to 60 h (Figure 4) following the degradation of red pigments in the pericarp (Wang et al., 2010). Moreover, although ANT degrades anthocyanins, we did not observe a clear correlation between ANT activity and protection against browning. In our study, we observed a sharp decrease in ANT activity for fruit inoculated with strain SI17 at 36–48 h and with strain PP19 at 24–36 h and 60–84 h (Figure 4). These results demonstrate that BCA treatment leads to comparable levels of enzymatic browning factors in treated and control fruit, results in higher defense responses, and subsequently leads to a lower incidence of fruit decay and browning in harvested fruit (Jiang et al., 2001; Cai et al., 2008; Wu et al., 2017).

## Conclusion

In this study, we showed that the four BCAs *B. amyloliquefaciens* PP19 and LI24, *E. acetylicum* SI17, and *B. licheniformis* HS10 are effective in controlling postharvest fruit browning in LTs, while strain SI17 also displayed high efficacy in FLT. Fruit browning inhibition of BCAs may rely on their colonization of litchi fruit, along with their effects on the enzymatic activity of antioxidants and the contents of relevant compounds. This study is the first report suggesting *E. acetylicum* strain SI17 as a promising BCA against litchi browning. In the future, we hope to design optimal combinations of preharvest and postharvest treatments, such as preharvest spraying with SI17 and postharvest spraying with PP19/SI17/HS10/LI24, which would be useful for developing sustainable management approaches to prevent fruit browning that minimize the use of chemical anti-browning agents and improve fruit shelf life.

## Data availability statement

The original contributions presented in the study are included in the article/Supplementary material, further inquiries can be directed to the corresponding authors.

## Author contributions

LZ and SH conducted the experiments and wrote the manuscript. LZ, SH, and JH analyzed the data. YD and ZW supervised the experiments and revised the manuscript. ZJ and GY designed the experiments and revised the manuscript. All authors contributed to the article and approved the submitted version.

## Funding

This research was supported by the earmarked fund for China Agriculture Research System (CARS-32), Guangdong Basic and Applied Basic Research Foundations (2020A1515110358), Key Laboratory of Biology and Genetic Resources Utilization of South Tropical Fruit Trees, Ministry of Agriculture and Rural Affairs (202102), and Guangdong University Key Laboratory for Sustainable Control of Fruit and Vegetable Diseases and Pests (KA21031C5).

## Conflict of interest

The authors declare that this research was conducted in the absence of any commercial or financial relationships that could be construed as a potential conflict of interest.

## Publisher’s note

All claims expressed in this article are solely those of the authors and do not necessarily represent those of their affiliated organizations, or those of the publisher, the editors and the reviewers. Any product that may be evaluated in this article, or claim that may be made by its manufacturer, is not guaranteed or endorsed by the publisher.
