# Supplementary material for: Biological control agents colonize litchi fruit during storage and stimulate physiological responses to delay pericarp browning
Source: Front Microbiol. 2023 Jan 5;13:1093699. doi: 10.3389/fmicb.2022.1093699 (PMC9849669; doi:10.3389/fmicb.2022.1093699)
Supplement: Supplementary file 1 [file Data_Sheet_1.docx]

Supplementary Material

**Supplementary Table S1 AE of strains four isolates in all LTs and FLT**

| Treatment | AE (%)x | | |
| --- | --- | --- | --- |
|  | LT 2017-1 | LT 2017-2 | FLT 2017 |
| PP19 | 30.1 | 60.6 | 12.5 |
| SI17 | 22 | 44.6 | 69 |
| LI24 | 32.3 | 35.2 | 34.3 |
| HS10 | 42.4 | 40.4 | -60.1 |
| BHT | 90 |  | 67.1 |

^x^ AE, stand for the average efficacy of a strain in the trials at every time point.

**Supplementary Table S2 AE and ARE of strains five isolates in in one LT and FLT**

| Treatment | Efficacy | | | | Percentage of the increase in AE and ARE with FLT relative to that with LT (%)^Z^ | |
| --- | --- | --- | --- | --- | --- | --- |
|  | LT2017-1 | | FLT2017 | |  |  |
| Strain | AE^x^ | ARE^y^ | AE | ARE | AE increase (%) | ARE increase (%) |
| PP19 | 30.1 | 33.5 | 12.5 | 18.7 | -58.4 | -44.2 |
| SI17 | 22 | 24.4 | 69 | 102.8 | 214.2 | 321.3 |
| LI24 | 32.3 | 35.9 | 34.3 | 51.1 | 6 | 42.3 |
| HS10 | 42.4 | 47.2 | -60.1 | -89.5 | -241.5 | -289.6 |
| BHT | 90 | 100 | 62 | 100 | -31.2 | 0 |

^x^ AE stand for the average efficacy of a strain in the trials; ^y^ ARE stands for average relative efficacy, represent the corresponding values relative to those of the BHT; ^y^ AE and ARE Increase (%) = (AE and ARE^FLT^ － AE and ARE^LT^) / AE and ARE^LT^ ×100%.


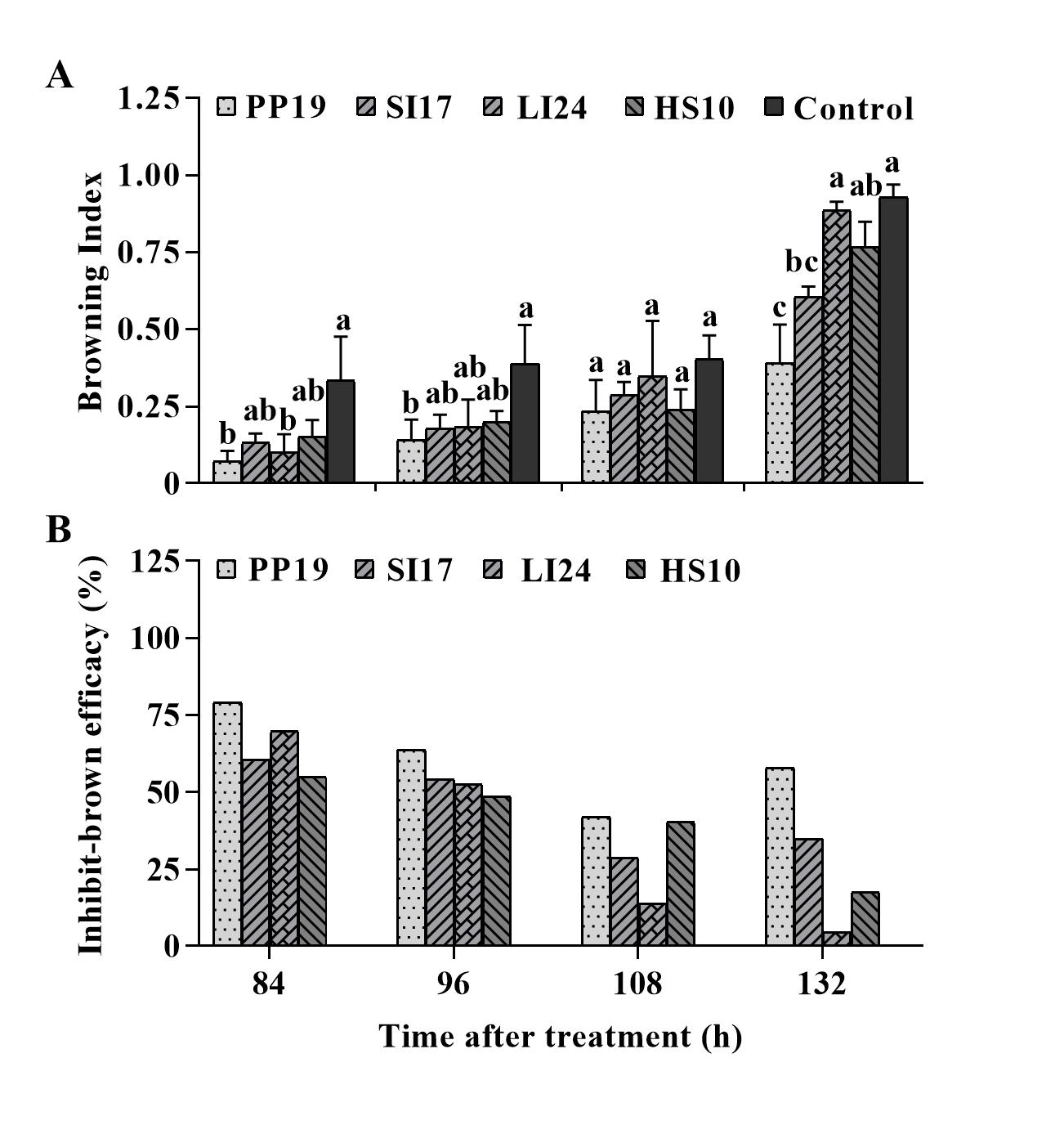


**Supplementary Figure S1 Inhibit-brown efficacy of litchi by four bacteria to *in vivo* fruit in LT2017-2.** Bacteria (5×10^7^ CFU/mL) were applied on cv. ‘Feizixiao’ (85 % ripening degree, Guangzhou, Guangdong province) as spraying in the laboratory. Browning index of harvested litchi fruit treated with the four isolates PP19, SI17, LI24, HS10 and the control of LB broth (A), and Inhibit-brown efficacy (B). Data are presented as means of replicates ± standard errors; different letters indicate significant differences between treatments according to LSD test at P < 0.05.
